# Gut Microbiota Diversity and C-Reactive Protein Are Predictors of Disease Severity in COVID-19 Patients

**DOI:** 10.3389/fmicb.2021.705020

**Published:** 2021-07-19

**Authors:** André Moreira-Rosário, Cláudia Marques, Hélder Pinheiro, João Ricardo Araújo, Pedro Ribeiro, Rita Rocha, Inês Mota, Diogo Pestana, Rita Ribeiro, Ana Pereira, Maria José de Sousa, José Pereira-Leal, José de Sousa, Juliana Morais, Diana Teixeira, Júlio César Rocha, Marta Silvestre, Nuno Príncipe, Nuno Gatta, José Amado, Lurdes Santos, Fernando Maltez, Ana Boquinhas, Germano de Sousa, Nuno Germano, Gonçalo Sarmento, Cristina Granja, Pedro Póvoa, Ana Faria, Conceição Calhau

**Affiliations:** ^1^Faculdade de Ciências M dicas, NOVA Medical School, Universidade NOVA de Lisboa, Lisbon, Portugal; ^2^Faculdade de Ciências Médicas, CINTESIS – Center for Health Technology and Services Research, NOVA Medical School, Universidade NOVA de Lisboa, Lisboa, Portugal; ^3^Department of Infectious Diseases, Hospital Curry Cabral, Centro Hospitalar Universitário de Lisboa Central, Lisboa, Portugal; ^4^Centro de Medicina Laboratorial Germano de Sousa, Lisboa, Portugal; ^5^i3S – Instituto de Investigação e Inovação em Saúde, Universidade do Porto, Porto, Portugal; ^6^Instituto de Biologia Molecular e Celular (IBMC), Universidade do Porto, Porto, Portugal; ^7^Ophiomics – Precision Medicine, Lisboa, Portugal; ^8^Faculdade de Ciências Médicas, Comprehensive Health Research Centre (CHRC), NOVA Medical School, Universidade NOVA de Lisboa, Lisboa, Portugal; ^9^Department of Emergency and Intensive Care Medicine, Centro Hospitalar Universitário de São João, Porto, Portugal; ^10^Infectious Diseases Service, ID Intensive Care Unit, Faculdade de Medicina, Centro Hospitalar Universitário de São João, Universidade do Porto, Porto, Portugal; ^11^Department of Emergency, CUF Infante Santo Hospital, Lisboa, Portugal; ^12^Polyvalent Intensive Care Unit, Hospital Curry Cabral, Centro Hospitalar Universitário de Lisboa Central, Lisboa, Portugal; ^13^Department of Internal Medicine, Centro Hospitalar de Entre o Douro e Vouga, Santa Maria da Feira, Portugal; ^14^Department of Anesthesiology, Centro Hospitalar Universitário de São João, Porto, Portugal; ^15^Department of Surgery and Physiology, Faculdade de Medicina, Universidade do Porto, Porto, Portugal; ^16^Polyvalent Intensive Care Unit, Hospital São Francisco Xavier, Centro Hospitalar Lisboa Ocidental, Lisboa, Portugal; ^17^Center for Clinical Epidemiology, Research Unit of Clinical Epidemiology, OUH Odense University Hospital, Odense, Denmark

**Keywords:** COVID-19, WHO Clinical Progression Scale, Shannon—Weiner diversity index, dysbiosis, gut microbiota

## Abstract

The risk factors for coronavirus disease 2019 (COVID-19) severity are still poorly understood. Considering the pivotal role of the gut microbiota on host immune and inflammatory functions, we investigated the association between changes in the gut microbiota composition and the clinical severity of COVID-19. We conducted a multicenter cross-sectional study prospectively enrolling 115 COVID-19 patients categorized according to: (1) the WHO Clinical Progression Scale—mild, 19 (16.5%); moderate, 37 (32.2%); or severe, 59 (51.3%), and (2) the location of recovery from COVID-19—ambulatory, 14 (household isolation, 12.2%); hospitalized in ward, 40 (34.8%); or hospitalized in the intensive care unit, 61 (53.0%). Gut microbiota analysis was performed through 16S rRNA gene sequencing, and the data obtained were further related to the clinical parameters of COVID-19 patients. The risk factors for COVID-19 severity were identified by univariate and multivariable logistic regression models. In comparison to mild COVID-19 patients, the gut microbiota of moderate and severe patients have: (a) lower Firmicutes/Bacteroidetes ratio; (b) higher abundance of Proteobacteria; and (c) lower abundance of beneficial butyrate-producing bacteria such as the genera *Roseburia* and *Lachnospira*. Multivariable regression analysis showed that the Shannon diversity index [odds ratio (OR) = 2.85, 95% CI = 1.09–7.41, *p* = 0.032) and C-reactive protein (OR = 3.45, 95% CI = 1.33–8.91, *p* = 0.011) are risk factors for severe COVID-19 (a score of 6 or higher in the WHO Clinical Progression Scale). In conclusion, our results demonstrated that hospitalized patients with moderate and severe COVID-19 have microbial signatures of gut dysbiosis; for the first time, the gut microbiota diversity is pointed out as a prognostic biomarker of COVID-19 severity.

## Introduction

Coronavirus disease 2019 (COVID-19), caused by the novel severe acute respiratory syndrome coronavirus 2 (SARS-CoV-2) infection, is clinically diverse in terms of disease severity—ranging from absence of symptoms to mild, self-limiting respiratory illness (including the common cold), severe pneumonia, acute respiratory distress syndrome, and death (Chen et al., 2020). COVID-19-induced respiratory distress syndrome was described to be associated with exuberant inflammation, intense cytokine production (cytokine storm syndrome), and multi-organ dysfunction (Chen et al., 2020; Marini and Gattinoni, 2020). Although respiratory symptoms are the most commonly reported among COVID-19 patients, gastrointestinal symptoms are also likely between SARS-CoV-2-infected patients, indicating that the gastrointestinal tract is an infected organ as well (Mao et al., 2020). In consequence, SARS-CoV-2 is detected in the feces of some COVID-19 patients (Wu et al., 2020b; Guo et al., 2021; Zuo et al., 2021).

Although the risk groups for severe COVID-19 were identified as being primarily the elderly and individuals with comorbidities, such as hypertension and diabetes (Petrakis et al., 2020; Wang D, et al., 2020; Zhou et al., 2020), COVID-19 may evolve adversely even in individuals without comorbidities, causing severe pneumonia, long-term sequelae, and, eventually, death (WHO, 2020). These observations suggest the existence of major predisposition factor(s) related to disease progression that need(s) to be urgently unveiled.

The human gut microbiota, mainly composed of bacteria, plays a critical role in health and most notably in host immune response, including vaccine efficacy (Ciabattini et al., 2019; Vlasova et al., 2019). Changes in the gut microbiota composition have been reported to affect both vulnerability and disease outcomes in non-communicable diseases, such as diabetes, inflammatory bowel disease, and obesity, leading to a state of chronic low-grade inflammation (Leocadio et al., 2019; Noce et al., 2019; Finlay et al., 2020). This role of the gut microbiota in both immune and inflammatory responses, together with the fact that SARS-CoV-2 binds to angiotensin-converting enzyme (ACE) 2 receptors on the gut epithelium (Ye et al., 2020), where it has been detected along the feces of COVID-19 patients (Xiao et al., 2020), suggests the existence of a microbial fingerprinting among these patients that may provide a predictive value for disease severity. Accordingly, gut microbiome characterization has been assessed in COVID-19 patients, which unveiled profound alterations on bacterial composition (Zuo et al., 2020, 2021; Yeoh et al., 2021). The depletion of beneficial bacteria from the taxa Lachnospiraceae and the genera *Bifidobacterium*, *Faecalibacterium*, and *Roseburia* (Gu et al., 2020; Zuo et al., 2020; Yeoh et al., 2021) has been proposed as having an impact on the modulation of host immune response to SARS-CoV-2 infection and potentially influenced disease severity and outcomes (Yeoh et al., 2021). However, existing studies did not enroll COVID-19 patients representative of the different COVID-19 severity levels, lacking mainly patients with severe clinical manifestations. Most importantly, previous studies did not clarify whether the observed changes in microbiota composition are a common patient’s response to SARS-CoV-2 infection rather than directly involved in disease severity.

Taking this into consideration, we investigated the association between the gut microbiota and COVID-19 disease severity using a cohort of 115 patients stratified as asymptomatic/mild–moderate–severe according to the WHO Clinical Progression Scale. Considering that previous studies have shown no significant alterations in the gut microbiota during COVID-19 disease progression and even after SARS-CoV-2 clearance (Zuo et al., 2020; Yeoh et al., 2021), a single fecal sample was collected. The clinical variables and gut bacterial composition were compared between the COVID-19 severity groups. The role of antibiotic use was also addressed. To the best of our knowledge, this is the largest study assessing the gut microbiota composition in patients with COVID-19 and the first outside of China.

## Results

### Clinical Characteristics of COVID-19 Patients

A total of 115 adults (median age = 68 years, 63.5% males) with a laboratory-confirmed positive test for SARS-CoV-2 were included in our study (Table 1). More than half (65.7%) were overweight or obese, and regarding comorbidities, 45 patients (42.1%) had diabetes, 67 (62.0%) had hypertension, and 21 (19.6%) had chronic respiratory disease (Table 1). Concerning antibiotic exposure, 42 patients (38.9%) were administered with antibiotics at least once during the 6 months prior to COVID-19 diagnosis (Table 1) and 108 (85.2%) were administered antibiotics during the course of COVID-19. Previous chronic therapy is not associated with COVID-19 severity (*p* = 0.403; Table 1).

**TABLE 1:** Clinical characteristics of the COVID-19 patients.

Characteristic	Total	Mild disease (scores 1–3)	Moderate disease (scores 4–5)	Severe disease (scores 6–9)	*p*-value
		
	(*N* = 115)	(*N* = 19)	(*N* = 37)	(*N* = 59)	
Age, median (IQR) (years)	68.0 (52.0–76.0)	61.0 (40.0–73.0)	71.0 (52.0–79.0)	66.0 (53.0–76.0)	0.305^a^
Male sex, *n* (%)	73 (63.5)	6 (31.6)	24 (64.9)	43 (72.9)	**0.032**^b^
Overweight or obese, *n* (%)	69 (65.7)	7 (70.0)	24 (66.7)	38 (64.4)	0.749^b^
Smoker, *n* (%)	21 (19.8)	2 (18.2)	5 (13.9)	14 (23.7)	0.467^b^
Pneumonia SARS-CoV-2, *n* (%)	84 (83.2)	2 (25.0)	24 (70.6)	58 (98.3)	**<0.001**^b^
C-reactive protein, median (IQR) (mg/L)	72.0 (28.3–158.9)	32.2 (17.9–54.5)	63.5 (11.5–115.6)	96.8 (34,0–177.0)	0.063^a^
Coexisting conditions, *n* (%)					
Diabetes	45 (42.1)	2 (16.7)	14 (38.9)	29 (49.2)	0.099^b^
Hypertension	67 (62.0)	4 (33.3)	27 (73.0)	36 (61.0)	0.811^b^
Chronic respiratory disease	21 (19.6)	2 (16.7)	5 (13.9)	14 (23.7)	0.236^b^
Immunosuppression	11 (10.9)	1 (12.5)	4 (11.8)	6 (10.2)	0.783^b^
Hematological-oncological disease	9 (8.5)	2 (16.7)	3 (8.6)	4 (6.8)	0.479^b^
Medication history, *n* (%)					
Previous chronic therapy	86 (86.9)	8 (100.0)	29 (87.9)	49 (84.5)	0.403^b^
Antibiotic therapy (last 6 months)	42 (38.9)	5 (41.7)	17 (45.9)	20 (33.9)	0.243^b^

Patients were classified in accordance with the WHO Clinical Progression Scale. This scale provides a measure of illness severity across a range from 0 (not infected with SARS-CoV-2) to 10 (dead). Patients were grouped into three categories: mild disease (scores 1–3), moderate disease (scores 4–5), and severe disease (scores 6–9). IQR, interquartile range ^a^Kruskal–Wallis test. ^b^Chi-square test. Bold values indicate statistical significance with a *p < 0.05.

According to the location of recovery, the proportion of patients with diabetes attending the intensive care unit (ICU) was significantly higher than the proportion of patients with diabetes isolated in a ward or those ambulatory (31 vs. 14 patients, *p* < 0.05). Similarly, the proportion of patients presenting three simultaneous comorbidities (obesity, hypertension, and diabetes) was higher in ICU patients than in those isolated in the ward or ambulatory (22 vs. 5 patients, *p* < 0.05).

### Fecal Microbiota Profile According to COVID-19 Severity

**FIGURE 1:**
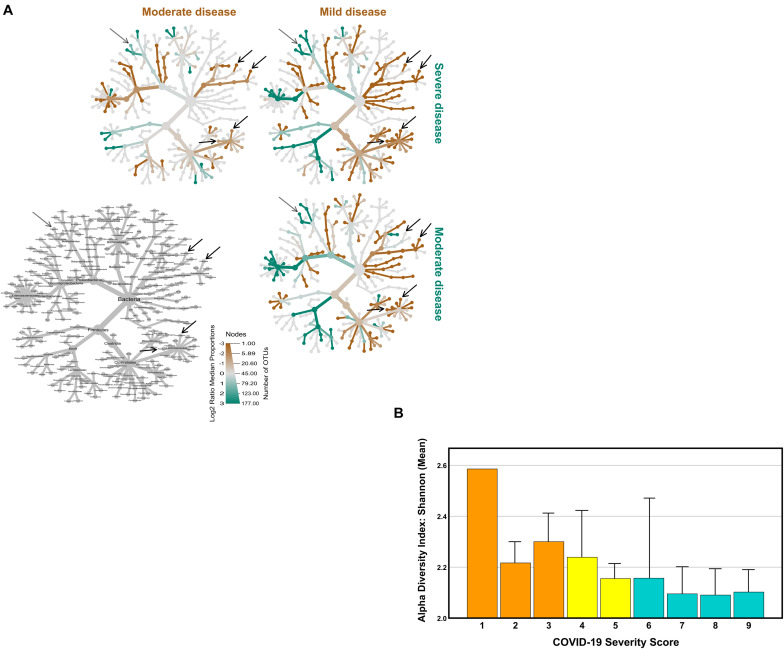
Comparison of coronavirus disease 2019 (COVID-19) gut microbiome with COVID-19 severity. Disease severity was determined according to the WHO Clinical Progression Scale: mild, moderate, and severe. **(A)** Heat tree visualization of the taxonomic differences between the COVID-19 severity groups based on the Log2 ratio median abundance (proportions), in which the *terminal nodes* correspond to bacterial genera. The identification of the nodes is shown in the *bottom left* image. Three comparisons were done: severe (*blue green*) vs. mild (*orange*); severe (*blue green*) vs. moderate (*orange*); and, ultimately, moderate (*blue green*) vs. mild (*orange*). The *dominant color* corresponds to a higher number of operational taxonomic units (OTUs). The Log2 ratio is 0 (*gray*) when the compared groups are similar. **(B)** The Shannon diversity index (mean + SEM) of the COVID-19 patients according to the WHO Clinical Progression Scale, from score 1 (asymptomatic, viral RNA detected) to score 9 (mechanical ventilation pO_2_/FiO_2_ < 150 and vasopressors, dialysis, or ECMO).

From the initial 115 COVID-19 patients, we were able to obtain a sufficient amount of good quality fecal DNA to perform microbial composition based on 16S ribosomal RNA (rRNA) gene analysis in 111 patients (96.5%). The gut microbiome of the COVID-19 patients was compared based on the fold change of relative abundance (medians) for each bacterial genus. For this comparison, the COVID-19 patients were grouped according to the disease severity defined by the WHO Clinical Progression Scale (WHO Working Group on the Clinical Characterisation and Management of Covid-19 infection, 2020). This scale provides a measure of illness severity, in which a higher score means a higher disease severity. Eighteen COVID-19 patients were classified as asymptomatic/mild (scores 1–3), 36 were categorized as moderate (4–5), while 57 were severe (scores 6–9). Three comparisons were done: (1) severe vs. asymptomatic/mild; (2) severe vs. moderate; and (3) moderate vs. asymptomatic/mild (hereinafter referred to as mild). In order to determine the relative taxonomic changes at the genus level between the COVID-19 severity groups, a heat tree was built for each comparison (Figure 1A), in which the terminal nodes correspond to the bacterial genera. For the first time, our data show that differences in the gut microbiome occur across all phyla, with the exception of Synergistetes and Verrucomicrobia, and the relative abundance is in general higher in less severe COVID-19 states. The higher number of alterations was observed between mild and moderate COVID-19 patients and between the mild and severe states. Fewer alterations were detected between the moderate and severe states of COVID-19. Globally, the relative abundances tend to be higher in mild than in moderate patients; in turn, the relative abundances tend to be higher in moderate than in severe COVID-19 patients. This decrease tendency from mild to moderate to severe is observed in the bacterial families Bifidobacteriaceae (*Bifidobacterium* genus) and Coriobacteriaceae (genus *Collinsella*), being statistically significant in the family Lachnospiraceae, namely, in the genera *Roseburia* and *Lachnospira* [*p* < 0.001, false discovery rate (FDR)-corrected]. In the opposite direction, the genus *Ralstonia* (Proteobacteria) increases with the COVID-19 severity score (*p* < 0.001, FDR-corrected).

In accordance with the inverse relation between the relative abundance of bacterial gut microbiota and COVID-19 severity score, Shannon’s diversity index showed a similar tendency, being higher in mild than in moderate and severe COVID-19 patients, with a mean values of 2.28 ± 0.30 (scores 1–3), 2.16 ± 0.40 (scores 4–5), and 2.10 ± 0.42 (scores 6–9), respectively (Figure 1B).

### Fecal Microbiota Profile According to COVID-19 Location of Recovery

As an indirect measure of the COVID-19 severity grade, the COVID-19 patients were grouped according with the location of recovery. Location of recovery and the WHO Clinical Progression Scale are related classifications; however, only the WHO scale provides a real measure of illness severity. Of the 111 COVID-19 patients with characterized fecal microbiota, 59 (53.2%) required ICU admission, 39 (35.1%) were hospitalized in a ward, and 13 (11.7%) were ambulatory (household isolation). Patient distribution by COVID-19 location of recovery (ambulatory–ward–ICU) does not totally overlap with the distribution by the WHO Clinical Progression Scale (mild–moderate–severe). There were some patients hospitalized in the ICU with moderate disease severity; also, there were other patients isolated at home with moderate symptoms.

**FIGURE 2:**
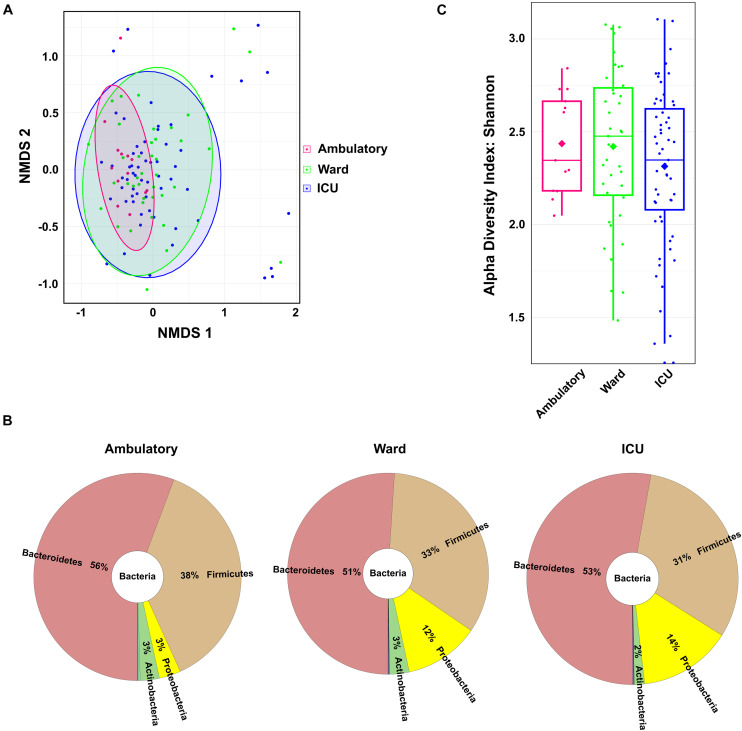
Fecal microbiota composition of coronavirus disease 2019 (COVID-19) patients according to patient location of recovery: ambulatory, hospitalized in ward, or hospitalized in ICU. **(A)** Fecal microbiota community alterations according to patient location in the NMDS2 (non-metric multidimensional scaling) plot based on the Bray–Curtis dissimilarity. **(B)** Main bacterial phyla in the fecal samples of COVID-19 patients according to patient location. **(C)** Box plot of the alpha diversity (measured by the Shannon diversity index) of the COVID-19 patients according to patient location.

The gut microbiome composition of all COVID-19 patients distributed by COVID-19 location of recovery was compared using the non-metric multidimensional scaling tool (Figure 2A). The fecal microbiota communities of COVID-19 patients recovering in ambulatory were more similar compared to the microbiota from those recovering in the ward and in the ICU [*p* < 0.05, permutational multivariate ANOVA (PERMANOVA)]. The comparison of the relative abundance at the phylum level between the three groups unveiled a consistent trend of an increase in the relative abundance of Proteobacteria from 3% in ambulatory patients to 12 and 14% in ward and ICU patients, respectively (Figure 2B). The Firmicutes/Bacteroidetes ratio decreased in COVID-19 patients from ambulatory–ward–ICU (0.68, 0.65, and 0.58, respectively). As observed for the WHO severity groups, the COVID-19 patients hospitalized in the ICU tended to have lower alpha diversity (Shannon’s index) in comparison to ambulatory and in ward/hospitalized COVID-19 patients (Figure 2C), as suggested by the lower mean and the first and third quartile values.

### Clinical Characteristics Associated With COVID-19 Severity

Univariate and multivariate logistic regression models were used to evaluate the associations between patients’ clinical characteristics and COVID-19 severity (Table 2). Our aim was to develop a prognostic model able in order to predict the occurrence of certain outcomes in severely vs. mild to moderately ill patients. The univariate model showed that severe COVID-19 patients were more likely to be men and to have elevated blood levels of C-reactive protein (CRP) compared with mild to moderate COVID-19 patients. The association between male gender and higher severity of COVID-19 can be explained by the higher proportion of men (72.9%) with severe COVID-19 in comparison to women. The age, body mass index, Shannon’s diversity index, comorbidities (hypertension and diabetes), and antibiotic therapy (at least once in the 6 months before COVID-19) were not significantly different between mild to moderate and severe patients. Regarding antibiotic therapy during the course of COVID-19, this variable was not significantly associated with COVID-19 severity (OR = 2.05, 95% CI = 0.55–7.73, *p* = 0.287).

**TABLE 2:** Bivariate logistic regression analysis of the clinical variables associated with severity of COVID-19 (a score of 6 or more in the WHO Clinical Progression Scale).

Variable		Crude^a^ OR^b^ (95% CI)	*p*-value	Adjusted^a^ OR^b^ (95% CI)	*p*-value
Gender	Female (*n* = 42)	1.0	**0.033**		
	Male (*n* = 73)	2.33 (1.07–5.07)			
Age	<65 year (*n* = 48)	1.0	0.603		
	≥65 (*n* = 67)	0.82 (0.39–1.73)			
C-reactive protein	<96.8 mg/L (*n* = 58)	1.0	**0.022**	1.0	**0.011**
	≥96.8 mg/L (*n* = 41)	2.73 (1.15–6.46)		3.45 (1.33–8.91)	
Shannon diversity index	≥2.25 (*n* = 46)	1.0	0.164	1.0	**0.032**
	<2.25 (*n* = 65)	1.72 (0.80–3.68)		2.85 (1.09–7.41)	
Overweight or obese	BMI < 25 (*n* = 36)	1.0	0.749		
	BMI ≥ 25 (*n* = 69)	0.88 (0.39–1.98)			
Hypertension	Normal (*n* = 41)	1.0	0.811		
	Hypertension (*n* = 67)	0.91 (0.42–1.99)			
Diabetes	Normal (*n* = 62)	1.0	0.101		
	Diabetes (*n* = 45)	1.93 (0.88–4.25)			
Antibiotic therapy (last 6 months)	Without (*n* = 66)	1.0	0.244		
	With (*n* = 42)	0.63 (0.29–1.37)			

95% CI, 95% confidence interval; OR odds ratio; BMI, body mass index. ^a^Crude ORs were calculated using univariate weighted logistic regression models. Adjusted ORs were calculated using multivariate weighted logistic regression models. Fully adjusted estimates take into account four variables (age, antibiotic therapy at least once in the last 6 months, C-reactive protein, and the Shannon diversity index) in the model (n = 96). ^b^Risk (OR) of severity of COVID-19 (a score of 6 or more in the WHO Clinical Progression Scale). Bold values indicate statistical significance with a *p < 0.05.

In the multivariate model mutually adjusted for CRP, Shannon’s diversity index, age, and antibiotic therapy 6 months prior to COVID-19 diagnosis, the variable CRP and Shannon’s diversity index were significantly associated with COVID-19 severity, while gender was no longer significantly associated (Table 2). Accordingly, the probability of having severe disease is 3.45 times higher when the CRP level is ≥ 96.8 mg/L. Likewise, the probability of having severe COVID-19 symptoms is 2.85 times higher when the Shannon diversity index is lower than 2.25. The geographic areas of the participating centers did not have an impact on our multivariate regression model, showing that disease severity and Shannon’s diversity index outcomes are center-independent. No correlation was found between the Shannon diversity index and clinical variables related to COVID-19 severity, such as duration of mechanical ventilation, ICU length of stay, ICU mortality, and 28-day mortality.

The discriminative/predictive power of the model was evaluated by receiver operating characteristic (ROC) curve analysis. The ROC analysis revealed an acceptable discriminative power of the model, with an area under the curve (AUC) of 0.707 (95% CI = 0.600–0.814) (Supplementary Figure 1). Furthermore, our model correctly predicted 56.4 and 78.9% of patients with mild to moderate and with severe disease, respectively.

### Fecal Microbiota Profile in Patients Positive for SARS-CoV-2 in Feces

**FIGURE 3:**
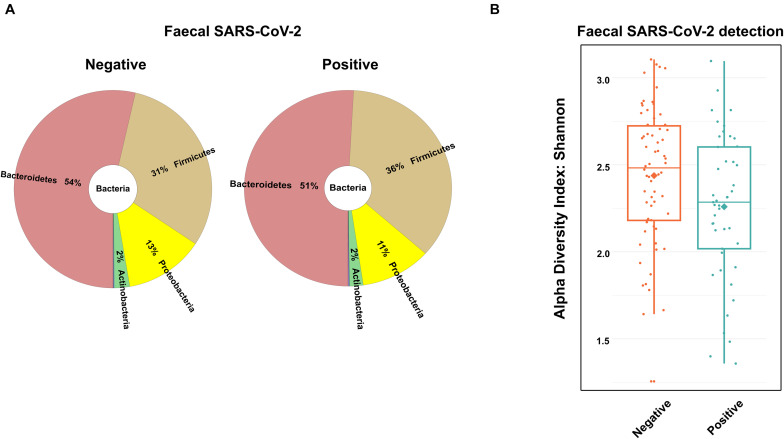
Fecal microbiota composition of the coronavirus disease 2019 (COVID-19) patients according to the presence of SARS-CoV-2 in fecal samples. **(A)** Main bacterial phyla and **(B)** box plot of the alpha diversity (measured by the Shannon diversity index) of COVID-19 patients according to the presence of SARS-CoV-2 in fecal samples.

With regard to some authors suggesting that fecal microbiota alterations are associated with the presence of SARS-CoV-2 in the gastrointestinal tract (Wolfel et al., 2020; Zuo et al., 2020; Yeoh et al., 2021), we analyzed the presence of SARS-CoV-2 RNA in feces. There was sufficient amount of good quality fecal RNA to detect SARS-CoV-2 RNA in 112 (97.4%) among the 115 recruited patients. From the 112 samples analyzed, 45 tested positive (40% of the COVID-19 patients). Interestingly, the virus was detected mostly in men than in women (61.3 and 38.7%, respectively, *p* < 0.05). We then investigated whether the presence of the virus in feces was associated with changes in the gut microbiota composition. As depicted in Figure 3A, no major differences were found in the distribution of the most abundant phyla and genera between patients positive and negative for SARS-CoV-2 in feces. Subsequently, we assessed the association between fecal SARS-CoV-2 positivity and the COVID-19 severity score or location of recovery using Pearson’s chi-square test. Importantly, no association was verified between the two categorical variables (*p*-values of 0.31 and 0.57 for the severity score and location of recovery, respectively). Nevertheless, we found a strong tendency for a lower Shannon’s diversity index in the feces of SARS-CoV-2 positive patients (*p* = 0.06; Figure 3B).

## Discussion

We conducted a multicenter prospective cross-sectional study with 115 COVID-19 patients in different COVID-19 severity stages under the hypothesis that gut microbiota dysbiosis plays a pivotal role in the pathophysiology of COVID-19, namely, in the severity of its clinical course.

**FIGURE 4:**
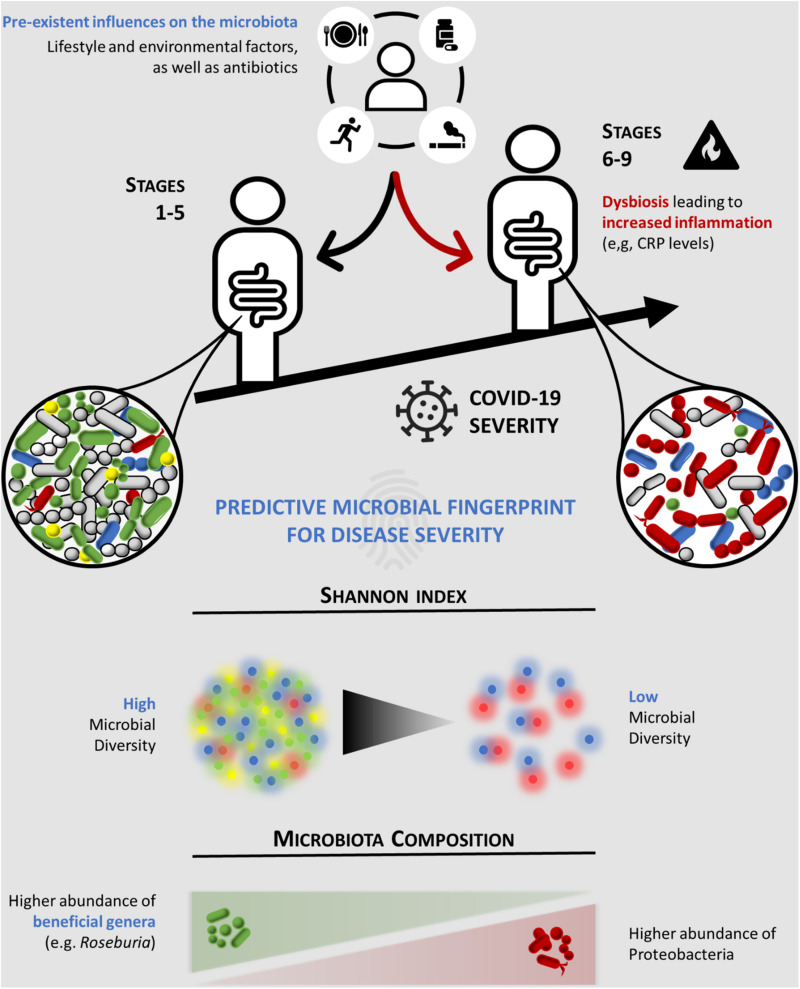
Schematic representation of the predictive microbial fingerprint for coronavirus disease 2019 (COVID-19) severity. Preexistent influences on the microbiota, such as lifestyle and environmental factors, and antibiotics can induce dysbiosis (*red arrow*), leading to increased inflammation (e.g., CRP levels). Hence, lower overall microbial diversity and abundance of beneficial commensal microorganisms (e.g., *Roseburia*), along with an increased abundance of Proteobacteria, are associated with high COVID-19 severity (a score of ≥ 6 in the WHO Clinical Progression Scale). CRP, C-reactive protein.

In order to determine the association between the gut microbiota composition and COVID-19 disease severity, the clinical and 16S rRNA gene sequencing data from COVID-19 patients were analyzed and subsequently clustered according to: (i) the severity of COVID-19 using the WHO Clinical Progression Scale, i.e., mild, moderate, or severe, and (ii) the location of recovery from COVID-19, i.e., ambulatory, hospitalized in ward, or hospitalized in ICU. Our data showed for the first time an inverse association between relative bacterial abundance at the genus level and Shannon’s index diversity with COVID-19 disease severity (Figure 4). According to our multivariable model, CRP ≥ 96.8 mg/L and a Shannon diversity index < 2.25 were associated with higher severity (a score of 6 or more in the COVID-19 WHO Clinical Progression Scale), suggesting that these patients’ variables are predictors of severe COVID-19. The data showed no correlation between the gut microbiota diversity (Shannon’s index) and the CRP levels. However, we cannot rule out a possible relation between the two biomarkers, and the possibility of crosstalk should be investigated in a larger study.

Our multivariable model correctly predicted 79% of patients with severe COVID-19. A cross-sectorial study including a larger population size is necessary to produce a more powerful multivariable logistic regression model that will predict a higher percentage of patients with severe COVID-19. Furthermore, other clinical variables that were not reported in this study could also contribute to improve the predictive and discriminative power of the model. Indeed, a recent study has demonstrated correlations between intestinal bacteria and interleukins, chemokines, and tumor necrosis factor-alpha, proposing that the gut microbiota could modulate the inflammatory immune response (Yeoh et al., 2021).

Interestingly, fecal SARS-CoV-2 was detected in COVID-19 patients who tend to have lower Shannon’s diversity (*p* = 0.06). We did not detect an association between fecal SARS-CoV-2 positivity and COVID-19 severity score (*p* = 0.31). However, this result should not be over-interpreted since a one-point fecal sample was collected, and thereby it cannot be excluded that patients with negative fecal SARS-CoV-2 could become positive during COVID-19 disease progression.

In comparison to mild COVID-19 patients, the gut microbiota from moderate and severe COVID-19 patients tended to have: (1) decreased Firmicutes/Bacteroidetes ratio (0.68 in mild compared to 0.65 and 0.58 in moderate and severe COVID-19, respectively); (2) higher abundance in Proteobacteria (3% in mild compared to 12 and 14% in moderate and severe COVID-19, respectively); (3) lower abundance of butyrate-producing bacteria from the family Lachnospiraceae, in particular the genera *Roseburia* and *Lachnospira*; and (4) lower abundance of the phylum Actinobacteria, namely, the genera *Bifidobacteria* and *Collinsella*. All these alterations are well-known microbial signatures of dysbiosis in the gut microbiota (Shin et al., 2015; Binda et al., 2018; Adelman et al., 2020; Magne et al., 2020). The Firmicutes/Bacteroidetes ratio has been used to evaluate gut microbiota dysbiosis; an increase or a decrease in this ratio is a hallmark of dysbiosis, the former being usually associated with obesity while the latter with inflammatory bowel disease (Stojanov et al., 2020). The observed modifications in the gut microbiota corresponded to a single-point fecal sample collection; therefore, we cannot exclude that other alterations can occur during COVID-19 disease progression.

Commensal bacteria play a fundamental role in the homeostasis of both the immune and inflammatory functions of the gut (Belkaid and Hand, 2014). Anaerobic bacteria from the family Lachnospiraceae, such as the genera *Roseburia* and *Lachnospira*, produce butyrate, a short-chain fatty acid known to exert anti-inflammatory effects in the intestinal epithelium (Segain et al., 2000). Despite not being butyrate producers themselves, *Bifidobacterium* species are able to cross-feed butyrate-producing bacteria through the secretion of fermentation end-products such as acetate (Riviere et al., 2016). This may constitute a potential mechanism by which *Bifidobacterium* species (Munoz et al., 2011; Li et al., 2016) counteract intestinal viral infections. Another mechanism might be related to their capacity to decrease the production of pro-inflammatory cytokines (e.g., tumor necrosis factor alpha and interferon gamma) and increase the production of anti-inflammatory cytokines (e.g., interleukins 4 and 10) (Sarkar and Mandal, 2016). Taking all these into consideration, we propose that changes in the gut microbiota composition observed in severe COVID-19 patients may eventually act as a trigger to promote mucosal inflammation and increased gut permeability to pro-inflammatory molecules. Consequently, this may induce a state of systemic inflammation since these patients exhibit higher levels of blood CRP, a recently recognized prognostic factor for COVID-19 severity (Wang G, et al., 2020). Likewise, a blood CRP concentration ≥ 96.8 mg/L is associated with a score of 6 or more in the COVID-19 WHO Clinical Progression Scale, in accordance with our multivariate model. The increase of Proteobacteria, a proposed signature of disease (Rizzatti et al., 2017) particularly of epithelial dysfunction (Litvak et al., 2017), in severe COVID-19 patients sustains our observation of a relation between microbiota dysbiosis and the severity of COVID-19 disease. Nevertheless, our observational study did not allow us to clarify whether the dysbiosis is directly involved in disease severity or could be a response to disease severity, despite an association between the gut microbiota and COVID-19 severity being demonstrated. The nature of this relation should be further explored.

Interestingly, men COVID-19 patients seemed more prone to severe disease when compared with women COVID-19 patients (*p* = 0.032). This gender discrepancy, which has been described in other clinical trials (Wu et al., 2020a), might be explained by the higher expression of ACE2 (Song et al., 2020) in intestinal epithelial cells. This protein receptor is required for SARS-CoV-2 binding, invasion, and persistence in host epithelial cells (Trottein and Sokol, 2020). Furthermore, COVID-19 patients who tested positive for the presence of SARS-CoV2 in feces were mostly men (*p* < 0.05), which reinforces the involvement of intestinal ACE2 in the severity of the course of the disease. The ACE2 receptor and the gut microbiota seem to be reciprocally regulated; some gut bacterial species seem to modulate colonic ACE2 expression (Geva-Zatorsky et al., 2017), while ACE2 regulates the production of antibacterial peptides through tryptophan transport, which, in turn, influences the gut microbiota composition (Hashimoto et al., 2012; Penninger et al., 2021).

Our findings are consistent with two previous cross-sectional studies with COVID-19 patients carried out in Hong Kong (China) (Zuo et al., 2020; Yeoh et al., 2021). The similarity of our results, collected in Portugal (a southwestern European country), to those of the geographically far distant Chinese population led us to conclude that gut microbiota dysbiosis is a bona fide predictor of COVID-19 severity, and the microbiome-based risk stratification should be considered in the management of SARS-CoV-2 infection susceptibility, in parallel with the worldwide-scale vaccination against COVID-19. Thus, our study opens perspectives for the development of therapeutic interventions that aim to correct dysbiosis in severe COVID-19 patients. These include dietary modifications, administration of butyrate-producing probiotics or prebiotics, and fecal microbiota transplantation from healthy donors (Friedland and Haribabu, 2020), shown to be effective in recurrent *Clostridium difficile* infection (Quraishi et al., 2017). These interventions are expected to increase the overall bacterial diversity and the abundance of commensal bacteria, thereby contributing to inhibit the overgrowth of bacteria from phylum Proteobacteria.

In summary, we revealed for the first time an association between the gut microbiota and the WHO Clinical Progression Scale, which reflects patient trajectory during COVID-19. Our data showed that gut microbiota dysbiosis is present in moderate and severe COVID-19 patients in comparison to asymptomatic/mild patients. Importantly, the evidence from this study suggests that CRP and gut microbiota diversity are prognostic biomarkers for severe COVID-19.

## Materials and Methods

### Study Design and Population

This national multicenter cross-sectional study was conducted in six geographically different Portuguese centers selected by invitation. The distributions of patients per participating center were 38 (33.0%), 33 (28.7%), 18 (15.7%), 12 (10.4%), 8 (7.0%), and 6 (5.2%). Patients’ eligibility criteria included age 18 years or above and a positive test for SARS-CoV-2 by nasopharyngeal swabs using quantitative RT-PCR performed in national reference laboratories and in accordance with the recommendations from the National Directorate of Health. COVID-19 patients were recruited during the first wave of the pandemic in Portugal—from April 21, 2020, to July 1, 2020—and sample size was determined based on the feasibility of recruitment during this period. The minimally detectable effect sizes were calculated retrospectively. In order to achieve a statistical power of 80% and a two-sided significance level of 0.05, and considering the total sample size of 115 individuals, the study was powered to detect a mean difference of 0.15 in the Shannon diversity index between mild to moderate and severe COVID-19 patients.

Participating centers prospectively collected data from consecutive patients included in the study and classified them according to the location of recovery (ambulatory and hospitalization in a ward or in the ICU) and the disease severity using the WHO Clinical Progression Scale (mild, moderate, and severe) (WHO Working Group on the Clinical Characterisation and Management of Covid-19 infection, 2020). The ethics committees and institutional review boards from the participating centers approved the study protocol, considering it a minimal-risk research using data collected for routine clinical practice, and waived the requirement to obtain informed consent. Patients (or their proxies) received written information about the study and were informed of their right to refuse to participate. The study was registered at ClinicalTrials.gov, no. NCT04355741. All authors had access to the study data and reviewed and approved the final version of the manuscript.

### Data Collection

Patient demographic characteristics, severity scores, smoking habits, comorbidities prior to hospitalization (diabetes, hypertension, chronic respiratory diseases, immunosuppression, hematological oncological disease, previous chronic therapy, and others), or antibiotic exposure 6 months prior to COVID-19 diagnosis were recorded for all patients at baseline (i.e., immediately after subject enrollment). Data on the clinical presentation of COVID-19, CRP levels, and the antibiotic, antiviral, and steroid treatments received during the course of the disease, as well as nutritional and respiratory support (as per the WHO Clinical Progression Scale) (WHO Working Group, 2020), were collected. In addition, clinical outcomes such as the duration of mechanical ventilation, ICU length of stay, ICU mortality, and 28-day mortality were also collected. Patients were followed up until hospital discharge, if that was the case.

### Stool Collection

Fecal samples of COVID-19 patients were collected after subject enrollment (single-point collection). The samples were collected with a stool collection kit (EasySampler, ALPCO, Salem, NH, United States) containing RNAlater (Sigma-Aldrich, St. Louis, MO, United States). The fecal samples were kept at −80°C until nucleic acid extraction.

### Gut Microbiota

Genomic DNA was extracted and purified from the stool samples of COVID-19 patients using the NZY Tissue gDNA Isolation Kit (NZYTech, Lisbon, Portugal). All 16S DNA libraries (V3 and V4 regions) were prepared, sequenced, and analyzed in accordance with the manufacturer’s instructions for each kit and instrument. Briefly, 16S DNA libraries were prepared using the Ion 16S^TM^ Metagenomics Kit targeted panel (Thermo Fisher Scientific, Waltham, MA, United States), and each sample was individually identified with the Ion Xpress^TM^ Barcode Adapters Kits (Thermo Fisher Scientific). All available regions were amplified using the Ion 16S^TM^ Metagenomics Kit (Thermo Fisher Scientific). The amplified fragments were then prepared for sequencing using the Ion CHEF System (Thermo Fisher Scientific) and loaded into Ion 318 Chip Kit v2 BC (Thermo Fisher Scientific). Sequencing runs were performed on an Ion S5 System (Thermo Fisher Scientific), aiming for a mean sequencing depth coverage of 12,000×. The sequencing depths were not normalized in order to achieve better identification of the alpha diversity in each sample. The sequencing data were filtered for length (cutadapt −m 80) and for quality (fastx_trimmer −l 280), after which the V3 and V4 regions were extracted (Mothur align.seqs and screen.seqs). The resulting fastq file was used for taxonomy. The taxonomy of each sample was determined using Kraken2^1^ and Bracken^2^ software, using our custom 16S database (GutHealth_DB). This database was manually curated by enriching GreenGenes (versions 13_5 and 13_8) with the clinically relevant taxa from NCBI RefSeq 16s rRNA sequences (04/2019). GutHealth_DB currently holds 4,765 16s rRNA sequences mapping 1,822 species, 1,685 genera, 515 families, 404 orders, 248 classes, and 89 phyla and is available upon request. Bacterial species were identified as pathogens or commensals according to The National Microbial Pathogen Database Resource (NMPDR)^3^. The datasets presented in this study can be found in the BioProject database^4^ with the accession number PRJNA734646.

### Detection of SARS-CoV-2 in Feces

The following steps were taken to detect SARS-CoV-2 in feces: (1) RNA extraction by the NucliSENS easyMAG technology based on the Boom technique that utilizes magnetic silica particles from 200 to 300 mg of stool and (b) detection of SARS-CoV-2 extracted RNA by the EURORealTime SARS-CoV-2 test. The latter is based on reverse transcription to convert viral RNA into complementary DNA, followed by PCR amplification and fluorescence-based real-time detection of two defined sections within the *ORF1ab* and *N* genes of the SARS-CoV-2 genome. Reverse transcription, amplification, and detection of SARS-CoV-2 cDNA were carried out by means of SARS-CoV-2-specific primers and probes.

### Statistical Analysis

Statistical analysis was performed using the SPSS version 27 software (SPSS Inc., Chicago, IL, United States) and R statistical software package V.3.5.1. Descriptive statistics are presented as numbers and percentages for categorical variables, as the mean and standard deviation (SD) for continuous variables, or as medians with interquartile ranges (IQRs) if the continuous variable is not normally distributed. Parametric (Student’s *t*-test and one-factor ANOVA) and non-parametric (Mann–Whitney and Kruskal–Wallis tests) tests were used as appropriate, taking into account normality assumptions and the number of groups compared. The Kolmogorov–Smirnov test was used to test normality assumptions of the variable distributions. Chi-square test and Fisher’s exact test were used as appropriate, for categorical variables.

Univariate and multivariate weighted logistic regression models were used in order to evaluate the risk factors associated with the severity of COVID-19 (a score of 6 or more in the WHO Clinical Progression Scale). The dependent variable in all models was the severity of COVID-19. Independent variables are indicated in the table legends (Table 2). The Hosmer–Lemeshow statistic and test was applied to evaluate the goodness of fit. The discriminative/predictive power of the model was evaluated by the ROC curve analysis. The influence of outlier data values on model fit was estimated using leverage statistics, and collinearity was assessed by evaluation of the coefficients’ correlation matrix. The results are presented as crude and adjusted ORs and their respective 95% confidence intervals. The statistical significance level was set at 5%, and differences were considered statistically significant when *p* < 0.05.

Heat tree visualization of the taxonomic differences between the COVID-19 severity groups was produced using the R package metacoder. Coloring indicates all differences between the median proportion of reads for the samples from patients grouped according to the severity of COVID-19 using the WHO Clinical Progression Scale, i.e., mild (scores 1–3), moderate (scores 4–5), and severe (scores 6–9) disease, as determined using a Wilcox rank-sum test followed by a Benjamini–Hochberg (FDR) correction for multiple testing.

Alpha diversity was measured by the Shannon diversity index that summarizes both the species richness (total number of species) and evenness (abundance distribution across species) within a sample. The distances (or dissimilarity) between samples of the same group were compared to the distances between groups using PERMANOVA.

### Missing Data Management

Considering that multiple imputation can give rise to biased results when missing data are not random (Hughes et al., 2019), regression analyses were based on complete data. In addition, a sensitivity analysis was performed using multiple imputations in order to account for missing data, with five imputed datasets and 10 iterations. All analysis results were aggregated with Rubin’s rule after appropriate transformation (Graham, 2020).

The sensitivity analysis in which the missing clinical variables were imputed by means of model-based multiple imputation showed similar results to the statistical analysis performed with complete cases (Shannon’s diversity index: OR = 2.71, 95% CI = 1.13–6.52, *p* = 0.026; CRP: OR = 4.42, 95% CI = 1.61–12.10, *p* = 0.004).

Since missing data were not equally distributed between the hospital datasets, we cannot ignore that missing data are not random. Since missing data at random assumption are not testable, we used complete case analysis as a better approach because multiple imputations could give rise to biased results. Nevertheless, a sensitivity analysis in which missing outcomes were imputed by multiple imputations was also carried out, and this analysis showed similar results, which suggests a limited effect of bias and strengthens the results obtained.

## Data Availability Statement

The data presented in the study are deposited in the BioProject repository, accession number PRJNA734646.

## Ethics Statement

The study was reviewed and approved by the ethic committee from Faculdade de Ci ncias Médicas, NOVA Medical School, Universidade NOVA de Lisboa, as well as by the ethic committees and institutional review boards from participating centers. The patients (or their proxies) provided their written informed consent to participate in this study.

## Author Contributions

AM-R, CM, PP, and CC contributed to the conception and design of the study. AM-R, CM, HP, JRA, RRo, IM, DP, MJS, JP-L, JM, DT, JR, AF, and CC interpreted the data. PR, RRi, AP, JS, and GSo analyzed and interpreted the data. HP, NP, NGa, JA, LS, FM, AB, NGe, GSa, CG, and PP acquired, analyzed, and interpreted the data. All authors contributed to the writing and editing of the article and approved the submitted version.

## Conflict of Interest

The authors declare that the research was conducted in the absence of any commercial or financial relationships that could be construed as a potential conflict of interest.

## Footnotes

1 https://ccb.jhu.edu/software/kraken2/

2 https://ccb.jhu.edu/software/bracken

3 https://www.patricbrc.org/view/Taxonomy/561#view_tab=genomes

4 http://www.ncbi.nlm.nih.gov/bioproject/

## Supplementary Material

Supplementary Figure 1 Receiver operating characteristics curve (ROC) analysis.
