# Supplementary figures and images for: Gut Microbiota Diversity and C-Reactive Protein Are Predictors of Disease Severity in COVID-19 Patients
Source: Front Microbiol. 2021 Jul 19;12:705020. doi: 10.3389/fmicb.2021.705020 (PMC8326578; doi:10.3389/fmicb.2021.705020)

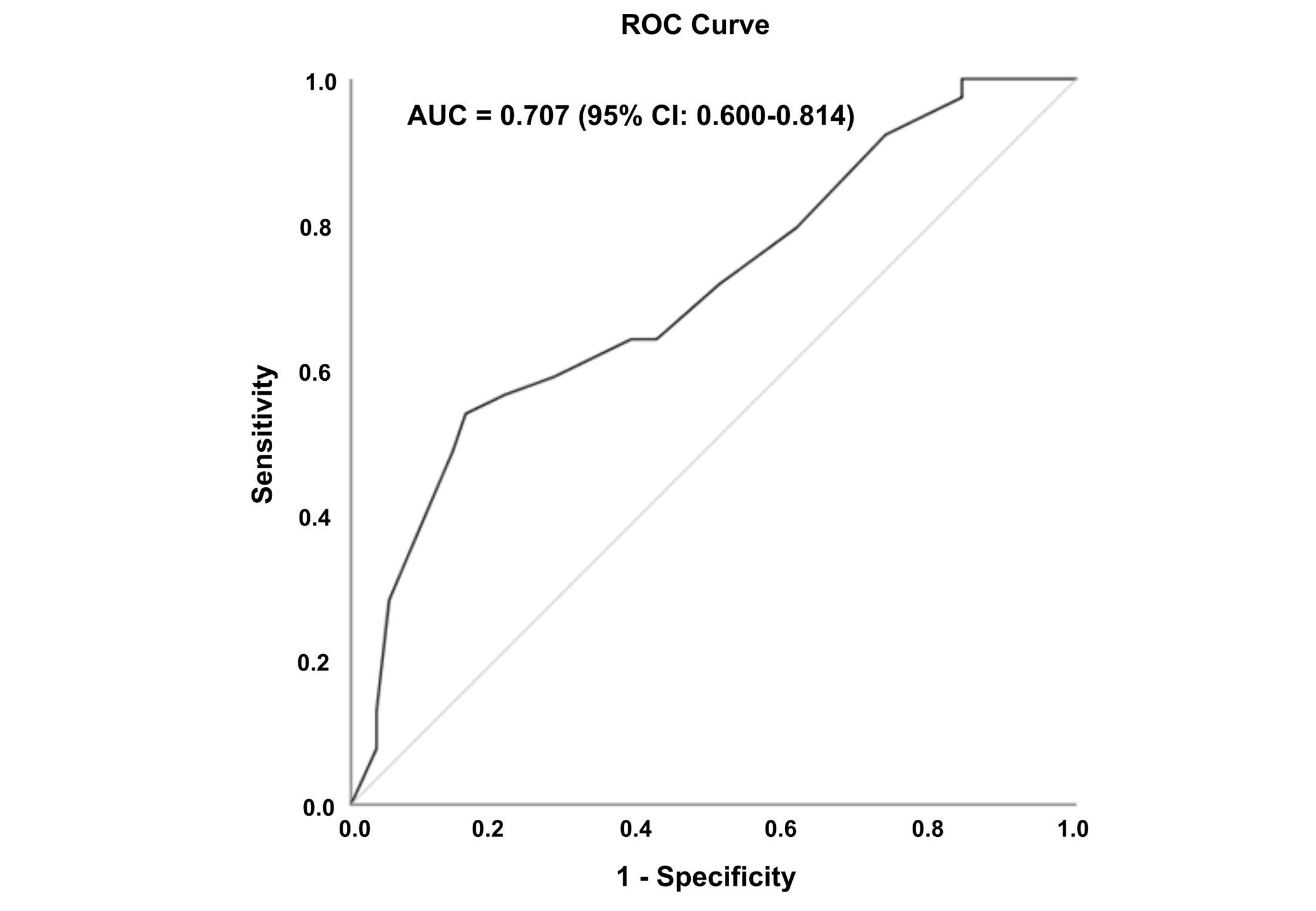

Supplement: Supplementary Figure 1 — Receiver operating characteristics curve (ROC) analysis. [file Image_1.TIF]
